# EAD-Net: A Novel Lesion Segmentation Method in Diabetic Retinopathy Using Neural Networks

**DOI:** 10.1155/2021/6482665

**Published:** 2021-09-01

**Authors:** Cheng Wan, Yingsi Chen, Han Li, Bo Zheng, Nan Chen, Weihua Yang, Chenghu Wang, Yan Li

**Affiliations:** ^1^Nanjing University of Aeronautics and Astronautics, College of Electronic and Information Engineering, 211106, China; ^2^Huzhou University, School of Information Engineering, 313000, China; ^3^The Affiliated Eye Hospital of Nanjing Medical University, 210029, China; ^4^The Affiliated Traditional Chinese Medicine Hospital of Southwest Medical University, 646000, China

## Abstract

Diabetic retinopathy (DR) is a common chronic fundus disease, which has four different kinds of microvessel structure and microvascular lesions: microaneurysms (MAs), hemorrhages (HEs), hard exudates, and soft exudates. Accurate detection and counting of them are a basic but important work. The manual annotation of these lesions is a labor-intensive task in clinical analysis. To solve the problem, we proposed a novel segmentation method for different lesions in DR. Our method is based on a convolutional neural network and can be divided into encoder module, attention module, and decoder module, so we refer it as EAD-Net. After normalization and augmentation, the fundus images were sent to the EAD-Net for automated feature extraction and pixel-wise label prediction. Given the evaluation metrics based on the matching degree between detected candidates and ground truth lesions, our method achieved sensitivity of 92.77%, specificity of 99.98%, and accuracy of 99.97% on the e_ophtha_EX dataset and comparable AUPR (Area under Precision-Recall curve) scores on IDRiD dataset. Moreover, the results on the local dataset also show that our EAD-Net has better performance than original U-net in most metrics, especially in the sensitivity and F1-score, with nearly ten percent improvement. The proposed EAD-Net is a novel method based on clinical DR diagnosis. It has satisfactory results on the segmentation of four different kinds of lesions. These effective segmentations have important clinical significance in the monitoring and diagnosis of DR.

## 1. Introduction

Diabetes is a common chronic disease that has a large number of patients over the world. It is a global public health problem related to microcirculation disorders which seriously affects human health. Diabetic retinopathy (DR) is a common complication of diabetes, so it is also a serious chronic disease. DR is caused by the insufficient blood supply and capillary occlusion due to excessive blood sugar content. In severe cases, it would lead to irreversible damage and even blindness. Therefore, the timely monitoring and treatment are essential for DR patients. The analysis of microvascular lesion areas is one of the important ways of diagnosis. In retinal fundus images, typical symptoms of DR mainly include microaneurysms (MAs), hemorrhages (HEs), hard exudates, and soft exudates, which are the major features of DR.

**Figure 1:**
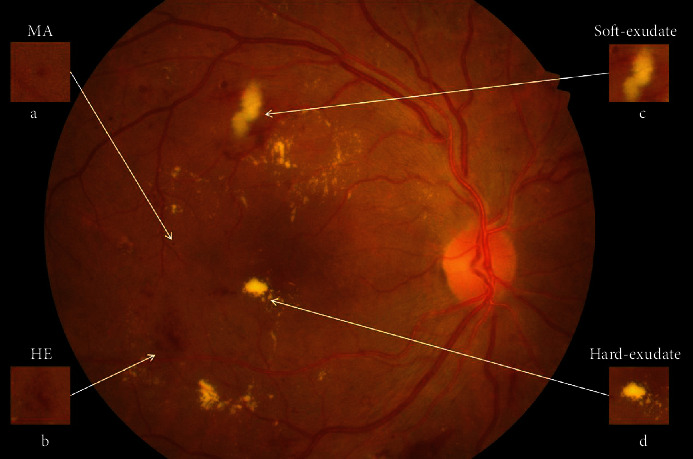
Lesions of DR in IDRiD_49.jpg from IDRiD dataset [1]. (a) Microaneurysm (MA). (b) Hemorrhage (HE). (c) Soft exudate. (d) Hard exudate.

As shown in Figure 1, the first detectable abnormalities of DR are MAs, which present as small red dots. MAs are formed due to the local distensions of capillary walls caused by high blood glucose on the surface of retina [2]. When MAs ruptured, they would cause intraretinal hemorrhages, which are also important features in the early stage of DR. HEs have irregular shapes and sizes, and their color is very similar to the background. Hard exudates are yellow lipid formations that leak as a result of increased capillary permeability, presenting as bright yellow exudates of irregular shape and well-defined boundaries. Soft exudates are essentially microinfarcts of the retinal nerve fiber layer, presenting as cotton-wool spots of irregular shape and fuzzy boundaries.

In recent years, many approaches have been applied to the lesion detection of DR, because detecting defective areas is an important step and one of the most labor-intensive tasks in clinical diagnosis. According to the characteristics of different lesion areas, we define the MAs and HEs as red lesions and define the hard exudates and soft exudates as bright lesions.

As for the detection of red lesions, since MAs and HEs are usually early abnormal signs of DR, the accurate detection of them is crucial for the early diagnosis. On account of similar circular shape and limited size range, the detections of MAs mainly include morphological operations [3, 4] and image filtering [5, 6]. Some other researches combine MA and HE detections, such as the algorithm based on curvelet transform proposed by Esmaeili et al. [7]. The candidate pixels belonging to red lesions and blood vessels are separated from a reconstructed retinal image with modified coefficients, and then, the full curvelet-based blood vessels are removed, leaving the remaining part as detected red lesions. Similarly, all dark-colored structures can be extracted as candidates, and then pixels belonging to vessels are eliminated by using a multilayer perception [8] or multiscale morphological closing operation [9]. However, the aforementioned algorithms might consider some actually red lesions as false positives to be removed, so these lesions are left out and directly affect the rate of detection. To solve the problem, an automatic red lesion detection algorithm using dynamic shape features [10, 11] is proposed. In this method, candidate regions do not need to be segmented precisely before feature extraction. Instead, a new set of shape features, called dynamic shape features, are extracted for each candidate region which is identified based on intensity and contrast.

As for the detection of bright lesions, Harangi and Hajdu [12] divided exudate detection into three stages: at first, a grayscale morphology-based candidate extractor method is used to recognize the bright lesions contained regions, then, an active contour method is applied to obtain the precise boundary segmentation, and finally, false exudate candidates are removed by a region-wise classifier. An unsupervised approach [13] for exudate segmentation is based on an ant colony optimization algorithm to solve the numerous manually labeling works needed in supervised methods. Many research works have been done for the detection of hard exudates: Banerjee and Kayal [14] proposed a method which employs morphological operations to eliminate optic disc, mean shift [15], and normalized cut [16] to extract hard exudates and Canny's operator to demarcate exudate boundary more clearly. Jaya et al. [17] proposed a hard exudate detection system designed using a fuzzy support vector machine (FSVM) classifier. In addition, only a few researches work for the detection of soft exudates (also called cotton wool spot) because it is difficult to filter out soft exudates from the background. Bui et al. [18] presented an automatic segmentation method which consists of image enhancement, optic disc removal, selective feature extraction, and a neural network model. Sreng et al. proposed an algorithm [19] based on the integration of principal component analysis (PCA) and support vector machine (SVM) for accurate detection of cotton wool spots. The authors also proposed another detection method [20] based on adaptive threshold and ant colony optimization (ACO) combined with SVM and achieved better performance.

With the development of convolutional neural networks (CNNs), various image segmentation algorithms have made breakthroughs in both speed and accuracy. One of the most popular methods for biomedical segmentation tasks was called U-net [21], which followed an encoder-decoder structure. There are many different improvements of the U-net model, such as an ensemble MU-net [22], designed to detect exudates with limited data, and a multitask architecture [23] for the joint segmentation of different lesions. Besides, Quellec et al. [24] proposed a deep learning algorithm supervised at image level and produced heatmaps to improve DR detection. Javidi et al. presented dictionary learning-based algorithms to segment exudates using extension of morphological component analysis [25] and to detect microaneurysm using sparse representation [26]. Dai et al. [27] combined an image-to-text model and multisieving CNN to identify microaneurysm and solve the unbalanced data distribution problem. Pratt et al. [28] also proposed a CNN approach for DR diagnosis and grading and achieved good performance on a large dataset.

The main contribution of this paper can be summarized as follows. Since the pixel-level lesion segmentation, especially, the segmentation of both red lesions and bright lesions is still rare, we focus on segmenting four different lesion areas with a supervised method which can work with limited labeled datasets. In this paper, we present a novel convolutional neural network EAD-Net, which is composed of encoder module, dual attention module, and decoder module. Experimental results show that the proposed EAD-Net can achieve pixel-level accuracy for different kinds of lesions. Our method has competitive performance in both qualitative and quantitative analyses than other state-of-the-art methods.

## 2. Methods

In this section, we describe the datasets used and the methods employed to segment different kinds of lesions. Firstly, in addition to two public benchmark datasets for the comparison with other state-of-the-art algorithms, a local dataset with hundreds of clinical images is also introduced for validation. Secondly, we describe the architecture of EAD-Net and illustrate the detailed structures of encoder module, dual attention module, and decoder module, respectively. Thirdly, we introduce the network training process including data normalization, data augmentation, and parameter settings. Finally, we designed an evaluation method based on the matching degree between detected candidates and ground truth lesions to analyze the segmentation results more appropriately.

### 2.1. Datasets

In this paper, we evaluated the performance of our proposed network on two publicly available datasets: e_ophtha_EX [29] and IDRiD [1], for the comparison with other latest algorithms. Furthermore, we also evaluated our model on a local intelligent ophthalmology dataset compared with U-net as the baseline for additional validation.

The public e_ophtha_EX dataset consists of 82 labeled images with precise lesion annotation. These images have four different sizes ranging from 1440 × 960 to 2544 × 1696 pixels. 47 images have exudates which were marked by two ophthalmologists, and 35 images contain no exudates.

The public IDRiD (Indian Diabetic Retinopathy Image Dataset) consists of 81 images with a resolution of 4288 × 2848 pixels. It provides pixel-level annotations of four lesions. The partition of the training set and testing set is provided on IDRiD, with 54 images for training and the rest 27 images for testing. All images in the testing set have MAs, HEs, and hard exudates, and 14 images of them have soft exudates.

**Figure 2:**
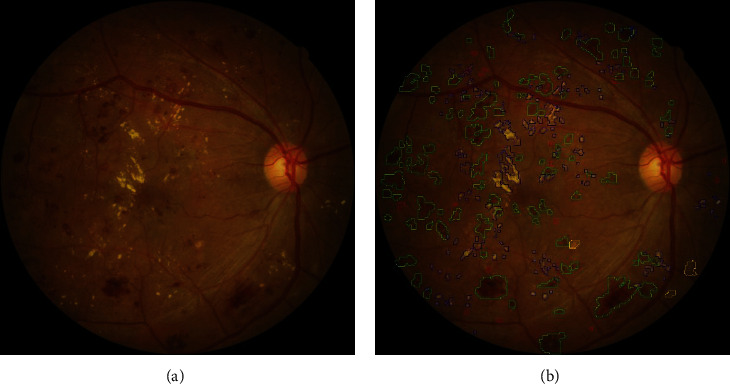
Annotation example of the local dataset. (a) The original image. (b) The corresponding annotation result: MAs in the red area, HEs in the green area, hard exudates in the blue area, and soft exudates in the yellow area.

The local intelligent ophthalmology dataset is a general high-quality dataset for eye disease classification and lesion segmentation. Our study was conducted in collaboration with the Affiliated Eye Hospital of Nanjing Medical University. From more than 10,000 clinical color fundus images, 262 images were selected for this research and all images have been desensitized for common use. In this dataset, 63 images have MAs, 84 images have HEs, 86 images have hard exudates, and 29 images have soft exudates. In addition, their corresponding pixel-level annotation images are provided. In lesion annotation, there were five ophthalmologists involved. To minimize the probability of mislabeling, all the images were labeled by four ophthalmologists and checked by a chief ophthalmologist at last. The detailed annotation example is shown in Figure 2.

### 2.2. Network Architecture

#### 2.2.1. Overview of the Proposed EAD-Net

**Figure 3:**
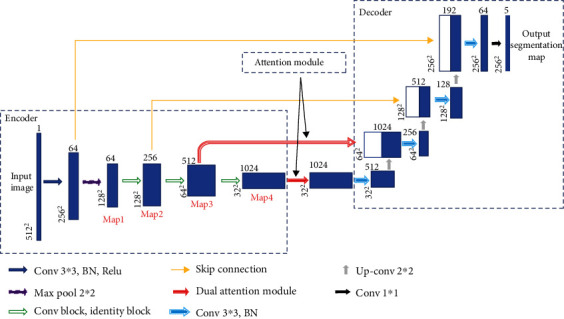
The overview of the EAD-Net architecture.

The proposed EAD-Net can be divided into three parts: encoder module, dual attention module, and decoder module (as shown in Figure 3). The U-shaped structure composed of an encoder and decoder, as well as skip connections, enables the network to combine high-level semantic information and low-level feature. Furthermore, the dual attention modules can capture long-range contextual information in both spatial and channel dimensions and therefore obtain better feature representations.

Specifically, through convolution and pooling, we can get the Map1; then, we use a convolution block with residual structure in the downsampling process. With the residual structure, the gradient can propagate directly through the skip connection from later layers to the earlier layers, so the vanishing gradient problem can be inhibited. These factors guarantee the stability of the whole network in a training process. Before the skip connection, Map3 and Map4 are sent through a dual attention module [30], which is composed of a position attention module and a channel attention module. Finally, the feature maps of each dimension are put into the decoder module to accomplish the segmentation of different kinds of lesions. Figure 3 shows an overview of the EAD-Net architecture.

#### 2.2.2. Encoder Module

**Figure 4:**
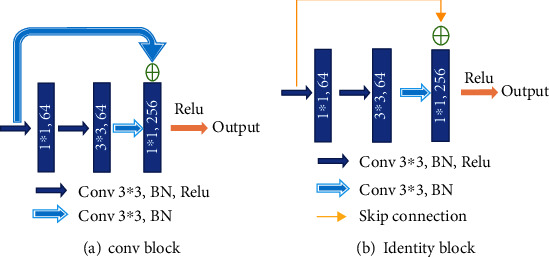
Detailed operations represented by the green arrow in the overview of the EAD-Net architecture.

Different from the widely used U-net, we choose a convolution block with a residual structure to replace the traditional encoder. And we only use the pooling layer once during the whole downsampling process. There are many tiny lesions in the segmentation of DR lesions, and too many pooling layers might go against recovering the features of the tiny targets in decoder stage. Therefore, in the later downsampling process, we use the convolution layer (stride is set to 2) to replace the pooling layer. The green hollow arrows in Figure 3 contain the conv block and identity block (as shown in Figure 4).

As we can see in Figure 4, conv block and identity block have almost the same structure. We learn from the idea of skip connection proposed in the ResNet [31]. In conv block, the input firstly passes through the same convolution, batch normalization, and Relu layers twice. Next, the result of the second Relu layer and the original input are added up after convolution and batch normalization. The added result is activated through the Relu layer to get the final output. What differentiates the two blocks is that in identity block the input is directly added up through a skip connection. One other thing to note is that the size of convolution kernels is set to the same 3 × 3.

The number of convolution kernels in the blocks shown in Figure 4 is subject to the bottleneck structure; that is, the output channel number of the input and output is generally four times as many as the channel number of the first two convolution parts. With this strategy, the number of training parameters can achieve a considerable reduction. It is worth noting that the number of channels indicated in the figure is not constant all the time. With the abovementioned proportional relationship, they will increase with the depending network, typically exponentially.

#### 2.2.3. Dual Attention Module

**Figure 5:**
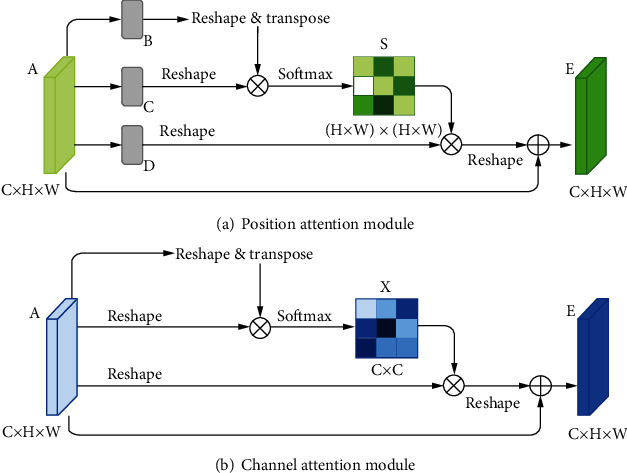
Illustration [30] of position attention module and channel attention module.

**Figure 6:**
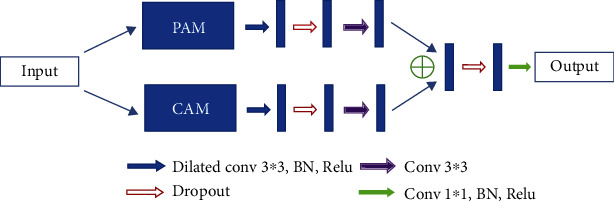
Detailed processing steps of dual attention module.

Dual attention module is a self-attention mechanism proposed by Fu et al. [30] and was applied to semantic segmentation. It can capture long-range contextual information in both spatial and channel dimensions. The position attention module (PAM) selectively aggregates the features of each position through a weighted sum of all positions, while the channel attention module (CAM) selectively emphasizes one feature map through all feature maps. The outputs of two attention modules are aggregated to obtain the better feature representations. The structure of dual attention module is shown in Figures 5 and 6.

In order to accommodate the specific morphology of DR lesions, we have also proposed some corresponding improvements.

There are often many small and fuzzy lesions existing in the fundus images. As what we have mentioned previously, too many pooling layers might lead to too much semantic information loss. To avoid this problem, we only use the max pooling layer once. Moreover, in the later decoder structure, to obtain the pixel-wise output, a larger size of feature map needs to be upsampled from the deep feature map, which might also cause the information loss. So the dilated convolution [32] strategy is introduced as an improvement. The dilated convolution with different dilation rates can produce a larger receptive field and capture multiscale contextual information. The blue sample block in Figure 6 contains three dilated convolutions. We set the dilation rates to 1, 2, and 5, respectively, to avoid gridding effect.

#### 2.2.4. Decoder Module

**Figure 7:**
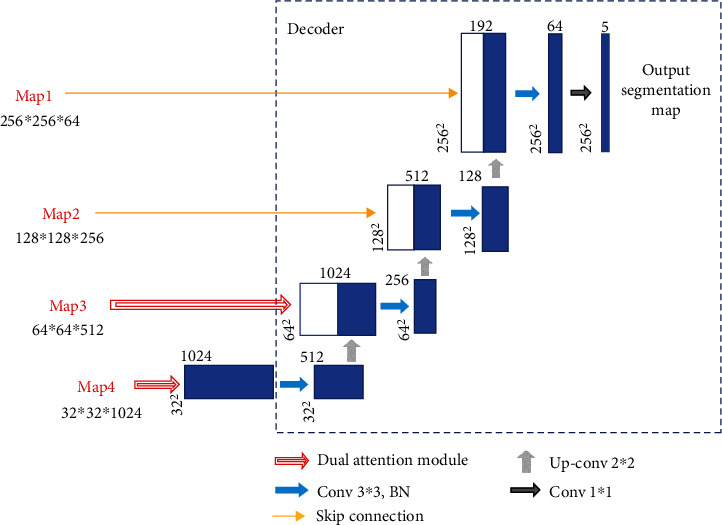
Illustration of the decoder module.

In the decoder module, we adopt the upsampling structure of U-net. The features of encoder and decoder at the same level can achieve global information fusion through concatenation. And the high-resolution information generated by the encoder output can provide more detailed guidance in the segmentation of lesions. The structure of decoder module is shown in Figure 7.

### 2.3. Network Training

**Figure 8:**
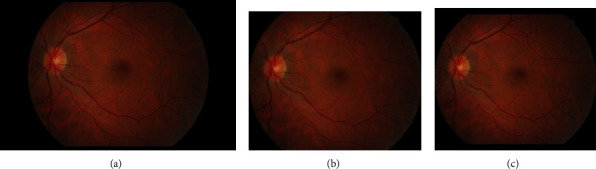
Size normalization process. (a) The original image. (b) Remove the redundant black edges. (c) The final result.

In order to facilitate the processing of neural network, the size of all input images and labels is normalized into 1024 × 1024 pixels. The purpose of this step is to preprocess the images and unify the size of all datasets without losing images' details. Meanwhile, in order to keep the information of input images as much as possible and make the image undistorted when its size changed (that is, maintain the aspect ratio of the image), we take the following steps: firstly, remove the redundant black edges around the original image. Next, according to the long side after the interception, the short side is filled to be equal to the long side. Finally, the size of the filled image is transformed to obtain an image of 1024 × 1024 pixels. We also cut and resize the corresponding ground truth segmentation image in the same way. The normalization process is shown in Figure 8.

High-quality datasets are valuable in the field of medical segmentation. Considering the lack of training data, data augmentation is beneficial when training the neural network. The data augmentation transformations consist of horizontally and vertically flipping, scaling images in per axis, translating, and rotation. Notice that we did not apply all these methods to every input image; instead, we select some combinations of them randomly to accomplish the augmentation. After data augmentation, the number of training dataset images could be up to five times larger.

Using the images with original size will run out of hardware limitations. In order not to lose image information in the maximum case, all images are resized to 512 × 512 pixels before being sent to the network training. Since the partition of training set and testing set is provided on IDRiD, with 54 images for training and the rest 27 images for testing, we also applied this partition ratio to e_ophtha_EX and local datasets in this research. There was not any overlap between training and testing data. For each dataset, two-thirds of the images were randomly selected for training and the remaining third for testing. That is to say, the partition ratio of training set and testing set is set to 2 : 1.

In the training process, firstly, the network's hyperparameters are gradually adjusted by the effect on the validation set. In this way, we set the batch size to 2, dropout rate to 0.5, Adam as the optimizer, and BCEDiceLoss (binary cross entropy and dice loss) as the loss function. In addition, we use the loss value as a monitoring indicator during training the network. The learning rate is set to 0.0001 and is lowered by 10 times after five epochs when the indicator does not improve. An early stopping method is also applied to the training process. If the indicator does not improve after 15 epochs, the training process would stop. The network for comparison follows the same training settings.

All the programs in this paper are based on Python. The construction and training process of the network are applied on Keras platform. Parallel computing is conducted by GPU, and the hardware environment is NVIDA GTX 1080.

### 2.4. Evaluation Metrics

**Figure 9:**
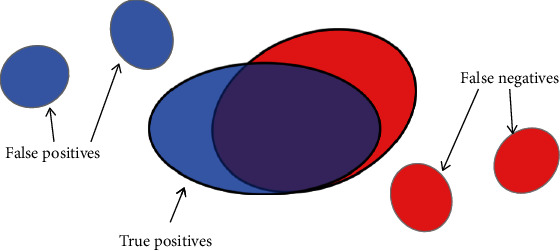
Illustration of the proposed evaluation method. Detected candidates are represented by blue areas and ground truth lesions by red areas. True positive pixels are defined by the matching degree of blue and red connected components.

The evaluation can be classically done by simply calculating the number of correctly identified pixels or by comparing the number of detected lesions with the number of real lesions. However, we consider that both of these methods have shortcomings in analyzing the segmentation of lesions. Suppose a situation as shown in Figure 9: there are three detected lesions (shown in blue) and three ground truth lesions (shown in red). Only the two large connected components in the middle are partially overlapped. It is clear that the larger the intersection area is, the greater the matching degree between the detected candidates and ground truth is.

On the one hand, if we only calculate the number of correctly identified pixels, in this case, the true positives only refer to the intersection area of blue and red, while half blue pixels and half red pixels in the nonoverlapping part are considered false positives and false negatives. This kind of evaluation method tends to get underestimated error rate on small connected components. On the other hand, it seems inappropriate to directly compare the number of detected lesions and ground truth lesions. For example, in Figure 9, there are 3 detected lesions and 3 ground truth lesions, but obviously, the results in the figure do not mean that the accuracy of lesion segmentation has reached 100%. Therefore, we applied the evaluation method proposed by Zhang et al. [29]: the matching degree between the detected candidates and the ground truth areas was considered. To be specific, if there are *N* detected candidates {*D*_1_, *D*_2_, ⋯, *D*_*N*_} and *M* ground truth lesions {*G*_1_, *G*_2_, ⋯, *G*_*M*_}, the set of detected candidates can be expressed as

(1) $$\begin{array}{c} D=\underset{1\leq i\leq N}{\cup }D_{\text{i}}, \end{array}$$

and the set of ground truth lesions can be expressed as

(2) $$\begin{array}{c} G=\underset{1\leq \text{j}\leq M}{\cup }G_{j}. \end{array}$$

Then we can give the definition of true positive (TP), false positive (FP), false negative (FN), and true negative (TN) as follows.

A pixel is considered TP if and only if it belongs to any of the following sets:

(i) (3) $$\begin{array}{c} D\cap G \end{array}$$
(ii) *D*_*i*_ such that (|*D*_*i*_∩*G*|/|*D*_*i*_|) > *σ*
(iii) *G*_*j*_ such that (|*G*_*j*_∩*D*|/|*G*_j_|) > *σ*

|·| is the cardinality of a set, and the *σ* is a factor used to evaluate the proportion of overlapping area between the detected candidates and ground truth. The *σ* ranges from 0 to 1. When *σ* = 0, a detected candidate is considered TP if and only if it touches the ground truth. Taking into consideration that in this case (*σ* = 0) a single very large detection mask would produce excellent results as long as it covers the whole ground truth set, a minimal overlap ratio is required. Finally, we set the *σ* to 0.2 to facilitate comparison with other methods.

A pixel is considered FP if and only if it belongs to any of the following sets:

- *D*_*i*_ such that *D*_i_∩*G* = *ϕ*
- $D_{i}\cap \bar{G}$ such that (|*D*_*i*_∩*G*|/|*D*_*i*_|) ≤ *σ*

A pixel is considered FN if and only if it belongs to any of the following sets:

(iii) *G*_j_ such that *G*_*j*_∩*D* = *ϕ*
(iv) $G_{j}\cap \bar{D}$ such that (|*G*_*j*_∩*D*|/|*G*_*j*_|) ≤ *σ*

Pixels that do not fall into any of the above-mentioned three categories are considered TN.

Then, we computed the sensitivity, specificity, precision, accuracy, and the F1-score according to the following equations:

(4) $$\begin{array}{c} \text{Sensitivity}=\frac{\text{TP}}{\text{TP}+\text{FN}},\\ \text{Specificity}=\frac{\text{TN}}{\text{TN}+\text{FP}},\\ \text{Precision}=\frac{\text{TP}}{\text{TP}+\text{FP}},\\ \text{Accuracy}=\frac{\text{TP}+\text{TN}}{\text{TP}+\text{TN}+\text{FP}+\text{FN}},\\ \text{F}1=\frac{2\times \text{sensitivity}\times \text{precision}}{\text{sensitivity}+\text{precision}}. \end{array}$$

## 3. Results

In this section, we demonstrate the effectiveness of our EAD-Net on two public benchmark datasets and show the comparison with other state-of-the-art algorithms, especially with U-net and its variants. For additional validation, we also compared the performance of our EAD-Net with the baseline U-net on a local dataset.

### 3.1. Performance on the Public e_ophtha_EX Dataset

On the public e_ophtha_EX dataset, the results compared with other state-of-the-art methods are shown in Table 1. Our proposed EAD-Net outperforms other methods on most indicators. Compared with the latest study [35] proposed by Guo et al., our method is 8.6% higher in sensitivity and achieves 5.61% and 7.06% improvements in precision and F1-score. Compared with the state-of-the-art method [22] by Zheng et al., our method has competitive results in both specificity and accuracy, although there exists a small gap in sensitivity, precision, and F1-score.

**Figure 10:**
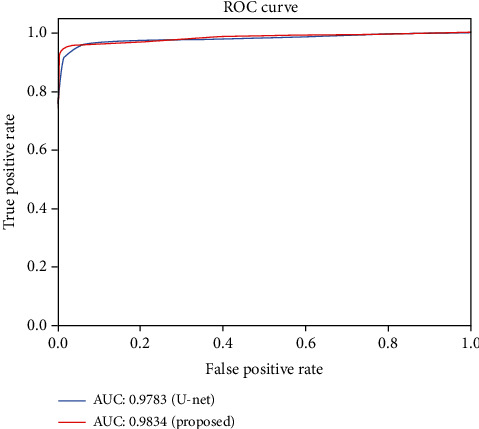
Evaluation on e_ophtha_EX dataset using ROC curves of U-net and the proposed EAD-Net.

Figure 10 also shows the ROC (Receiver Operating Characteristic) curves with AUC (Area Under Curve) values of our method and the baseline, U-net. We can see that the EAD-Net has much better detection effect than the original U-net. The AUC value of the proposed method is 0.5% higher than the result of U-net. The improved performance demonstrates the effectiveness of the proposed EAD-Net.

### 3.2. Performance on the Public IDRiD Dataset

In this part, we used AUPR (Area under Precision-Recall curve) as evaluation metric, which is the same to the IDRiD challenge. The IDRiD challenge is a fundus image analysis challenge organized by the IEEE International Symposium on Biomedical Imaging (ISBI) conference. We compared our method with the top 10 teams in the lesion segmentation competition of IDRiD challenge. As we can see in Table 2, the proposed EAD-Net ranked No. 3 on HE segmentation, No. 6 on hard exudate segmentation, and No. 4 on soft exudate segmentation.

For the top 3 teams, they choose different network architectures for each segmentation task. And for each segmentation task, many hyperparameters need to be adjusted during the training stage. Therefore, these teams that performed well had to test four models for corresponding segmentation task during the test stage. In contrast, our study used a single network structure and only a few changes are needed for the hyperparameter settings. Even so, our proposed EAD-Net has achieved comparable results.

### 3.3. Performance on the Local Intelligent Ophthalmology Dataset

On the local intelligent ophthalmology dataset, we also evaluated the performance by comparing the matching degree between the ground truth and prediction. In this section, we performed a visual analysis of segmentation results and compared our proposed method with the original U-net, which was the baseline.

**Figure 11:**
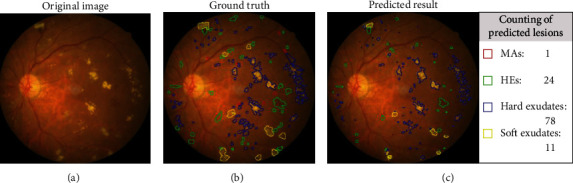
An example of segmentation results. (a) Original image. (b) The corresponding ground truth annotations of different lesions. (c) The predicted segmentation results. Note that the areas marked out in different colors represent different lesions: MAs in red, HEs in green, hard exudates in blue, and soft exudates in yellow. The predicted result in (c) also provides counting statistics of the four lesions. In the rectangular box on the right of (c), the number of lesions is obtained by connected components analysis of the corresponding lesion areas in the left of (c).

**Figure 12:**
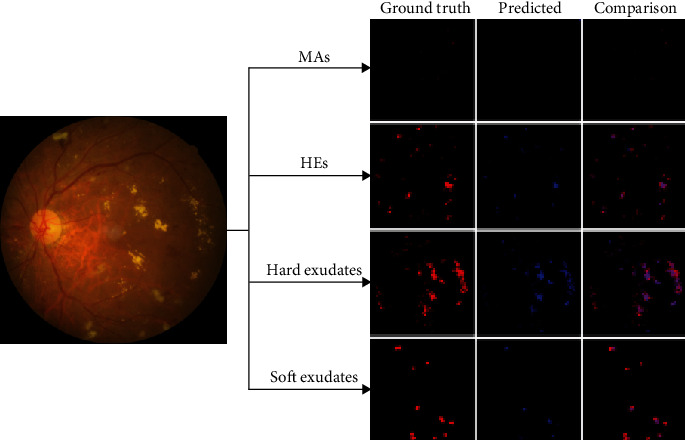
Detailed comparison of ground truth and predicted segmentations. Each row represents a kind of lesions. The last column represents the superimposed image of the first two images in the row. In red: ground truth areas; in blue: predicted lesions areas; in purple: the intersection of ground truth and prediction.

An example of segmentation results is shown in Figure 11: different color curves are used to represent the contours of different types of lesions. At the same time, through connected components analysis in the predicted images, we can also easily output the counting of different lesions (as shown in the rectangular box on the right of Figure 11(c)). These counting statistics are helpful as a reference for clinical diagnosis of DR severity. In addition, a more detailed comparison of ground truth and predicted segmentations for this example is shown in Figure 12. The different rows in Figure 12 represent different types of lesions. We use red to represent ground truth areas, blue to represent predicted lesions areas, and purple to represent the intersection of ground truth and prediction in the last column of Figure 12. From this, we can intuitively see which areas are correctly identified, which areas are misdiagnosed, and which areas are missed. For detailed definitions of the categories of predicted lesions (TP, FP, FN, or TN), please refer to Evaluation Metrics.

**Figure 13:**
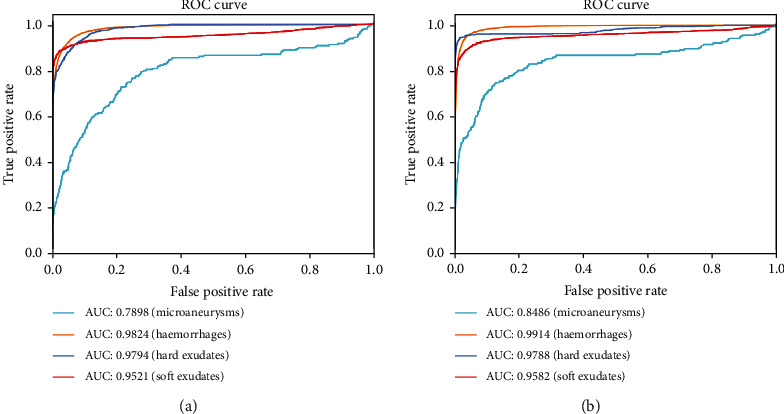
Evaluation on the local intelligent ophthalmology dataset. Different colored curves represent different types of lesions. (a) ROC curves of U-net. (b) ROC curves of EAD-Net.

Compared with the baseline U-net, the results shown in Table 3 indicate that the proposed method outperforms the original U-net in most metrics, especially in the sensitivity and F1-score. And the AUCs of the EAD-Net are generally higher than U-net (as shown in Figure 13).

From all the above results on the local dataset, it can be concluded that the EAD-Net makes remarkable progress in the lesion segmentation compared with baseline U-net. However, although our network does well in the segmentation of the lesions with distinct features, such as HEs and hard exudates, the details in Figure 12 and the low sensitivity in Table 3 indicate that it is not that effective for small lesions, especially the tiny MAs. This problem would be discussed in more detail in the next section.

## 4. Discussion

The research of computer-aided diagnosis of DR based on fundus images is an emerging field. Most of the current DR-AI researches are based on the image labels, rather than the direct study of lesions. However, the diagnosis basis of clinical guidelines is precisely based on the identification and localization of lesions. Once the clinical guidelines are adjusted, none of the current DR-AI results can play a role. In contrast, lesion-based studies can be easily adapted to the adjustment of diagnostic rules. Therefore, we proposed a deep learning method based directly on lesions, which is aimed at segmenting four typical lesions of DR: MAs, HEs, hard exudates, and soft exudates. In addition, the proposed method can easily output the counting of different lesions, so as to diagnose the severity of DR. In this paper, we designed a novel convolutional neural network named EAD-Net, which is composed of encoder module, dual attention module, and decoder module.

The proposed network has significant improvement in the segmentation of different lesions: MAs, HEs, hard exudates, and soft exudates. Different from the original U-net, we choose a convolution block with residual structure to replace the traditional encoder. Since there exist many small or fuzzy lesions and too many pooling layers might lead too much semantic information loss, we only use the max pooling layer once to avoid this problem. The dual attention module is designed to capture long-range contextual information in both spatial and channel dimensions, so that the network can obtain better feature representations. We also introduce the dilated convolution strategy as an improvement. By setting different dilation rates, we can get larger receptive field and multiscale contextual information. The high-resolution information generated by the encoder output can provide more detailed guidance in the segmentation of lesions.

Compared with other state-of-the-art methods, we achieve superior performance on two public benchmark datasets: e_ophtha_EX and IDRiD. As a variant of U-net, the proposed EAD-Net outperforms the baseline U-net at both lesion-level and image-level by a large margin. As an additional validation, the results on the local dataset also demonstrate the effectiveness of our method.

However, the drawback of EAD-Net is the limited detection performance for tiny lesions, such as MAs and small exudates. As an instance shown in Figure 11, there exist omissions and misidentifications of MAs, and some blood vessels are also detected as HEs. The reason might be that unlike natural images, medical images tend to be more complicated, and they are influenced by many factors, such as imaging equipment, and illumination effect. In the fundus images of DR, there exist many tiny and fuzzy lesions. It is not easy to find the boundary between these lesions and their adjacent pixels, and even professional doctors need a long time to locate them. To better analyze the experimental results, we calculated the distribution of labeled lesions in three datasets. The statistics information is shown in Table 4.

From Table 4, we can see that the ratio of MAs is very small, which makes it very difficult to accurately segment. However, since our study only used a single network structure, the drawback could be overcome by ensemble networks or more elaborate preprocessing in a further study.

In the three different datasets we used, there were 35 normal images and 47 abnormal images in the e_ophtha_EX dataset, while the 81 images in the IDRiD dataset and 262 images in our local intelligent ophthalmology dataset were all with more or less different lesions. To a certain degree, the performance of our method on e_ophtha_EX dataset can demonstrate its robustness to normal samples. Furthermore, the fundus images of three datasets we used were from people in different countries, which proved that the proposed method was robust to a certain extent for different ethnic groups. In further studies, we need to conduct experiments on a larger and more balanced data distribution to adapt to various situations in a real world.

## 5. Conclusion

The DR-AI research based directly on lesions is in line with clinical diagnostic thinking of ophthalmology. In this paper, we propose a convolutional neural network architecture EAD-Net for the lesion segmentation task. The architecture can be divided into three parts: encoder module, dual attention module, and decoder module. On both public and local datasets, we compare the performance of the EAD-Net with other state-of-the-art methods and prove its superiority. Experimental results show that our network has satisfactory results on the segmentation of four different kinds of lesions. These effective segmentation results have important clinical significance in the screening and diagnosis of DR. With more accurate performance and appropriate diagnostic rules based on the lesions, the proposed method will be more suitable for the clinical application.

## Figures and Tables

**Table 1:** Evaluation of exudate detection on e_ophtha_EX dataset.

Model	Lesion-level results				
	SE	SP	PR	ACC	F1
U-net	79.86	99.97	78.77	99.95	79.31
^∗^Playout et al. [23]	80.02	—	78.50	—	79.25
^∗^Zheng et al. [22]	94.12	99.98	91.25	99.96	92.66
Fraz et al. [33]	81.20	94.60	90.91	89.25	—
Zhang et al. [29]	74	—	72	—	—
Imani and Pourreza [34]	80.32	99.83	77.28	—	—
Javidi et al. [25]	80.51	99.84	77.30	—	—
Guo et al. [35]	84.17	—	83.45	—	83.81
^∗^Proposed EAD-Net	92.77	99.98	89.06	99.97	90.87

SE: sensitivity; SP: specificity; PR: precision; ACC: accuracy; F1: F1 score. ∗ are methods based on U-net.

**Table 2:** Comparison with top 10 teams in the lesion segmentation competition on IDRiD dataset.

Model (team)	MAs	HEs	Hard exudates	Soft exudates
VRT (1st)	0.4951	0.6804	0.7127	0.6995
PATech (2nd)	0.4740	0.6490	0.8850	—
iFLYTEK-MIG (3rd)	0.5017	0.5588	0.8741	0.6588
SOONER (4th)	0.4003	0.5395	0.7390	0.5369
SHAIST (5th)	—	—	0.8582	—
lzyuncc_fusion (6th)	—	—	0.8202	0.6259
SDNU (7th)	0.4111	0.4572	0.5018	0.5374
CIL (8th)	0.3920	0.4886	0.7554	0.5024
MedLabs (9th)	0.3397	0.3705	0.7863	0.2637
AIMIA (10th)	0.3792	0.3283	0.7662	0.2733
Proposed EAD-Net	0.2408	0.5649	0.7818	0.6083

The results are based on AUPR (Area under Precision-Recall curve).

**Table 3:** Comparison with U-net on local intelligent ophthalmology dataset.

Lesion type	Model	Lesion based results				
		SE	SP	PR	ACC	F1
MAs	U-net	13.17	99.97	54.07	99.90	21.19
	EAD-Net	17.32	99.98	59.26	99.91	26.82
HEs	U-net	73.43	99.93	80.21	99.83	76.67
	EAD-Net	83.59	99.95	87.75	99.89	85.62
Hard exudates	U-net	68.38	99.99	98.42	99.96	80.70
	EAD-Net	84.60	99.99	93.51	99.98	88.83
Soft exudates	U-net	76.89	99.99	98.86	99.98	86.50
	EAD-Net	84.92	99.99	92.78	99.98	88.68

SE: sensitivity; SP: specificity; PR: precision; ACC: accuracy; F1: F1 score.

**Table 4:** Statistical information of lesion areas of e-ophtha, IDRiD, and local intelligent ophthalmology datasets.

Dataset	MAs	HEs	Hard exudates	Soft exudates
E-ophtha	0.01% (148)	—	0.22% (47)	—
IDRiD	0.10% (81)	1.03% (80)	0.90% (81)	0.38% (40)
Local	0.02% (63)	0.91% (84)	0.48% (86)	0.32% (29)

Number1 (Number2) refers to the fact that there are Number2 images of this lesion type in the corresponding dataset, and the average percentage of this lesion area to the total image area is Number1. To maintain data consistency, only the images containing lesions have been used in Number1.

## Data Availability

Datasets of e_ophtha_EX and IDRiD used to support this study are available at doi:10.1016/j.media.2014.05.004 and doi:10.3390/data3030025. These prior studies (and datasets) are cited at relevant places within the text as references [1, 29]. The local intelligent ophthalmology dataset used to support the findings of this study is from the Affiliated Eye Hospital of Nanjing Medical University and is currently under embargo while the research findings are commercialized. Requests for data, 6 months after publication of this article, will be considered by the corresponding author.
